# Supplementary material for: CRISPR activation screen in mice identifies novel membrane proteins enhancing pulmonary metastatic colonisation
Source: Commun Biol. 2021 Mar 23;4:395. doi: 10.1038/s42003-021-01912-w (PMC7987976; doi:10.1038/s42003-021-01912-w)
Supplement: Supplementary file 2 — Supplementary Information [file 42003_2021_1912_MOESM2_ESM.pdf]

Supplementary Figure 1

a

SCREEN STATISTICS	PLASMID	CELLS (500x)	LUNG (4h)	LUNG (19d)
Total read count (median ± SD)	2,982,555 ± 0	3,715,146 ± 0.3M	6,855,366 ± 3.9M	4,668,658 ± 1.2M
Mean reads per gRNA	266.42 ± 0	331.86 ± 26	612.36 ± 352	417.03 ± 110
Non-mapping read fraction	0.23 ± 0	0.18 ± 0.06	0.18 ± 0.07	0.19 ± 0.09

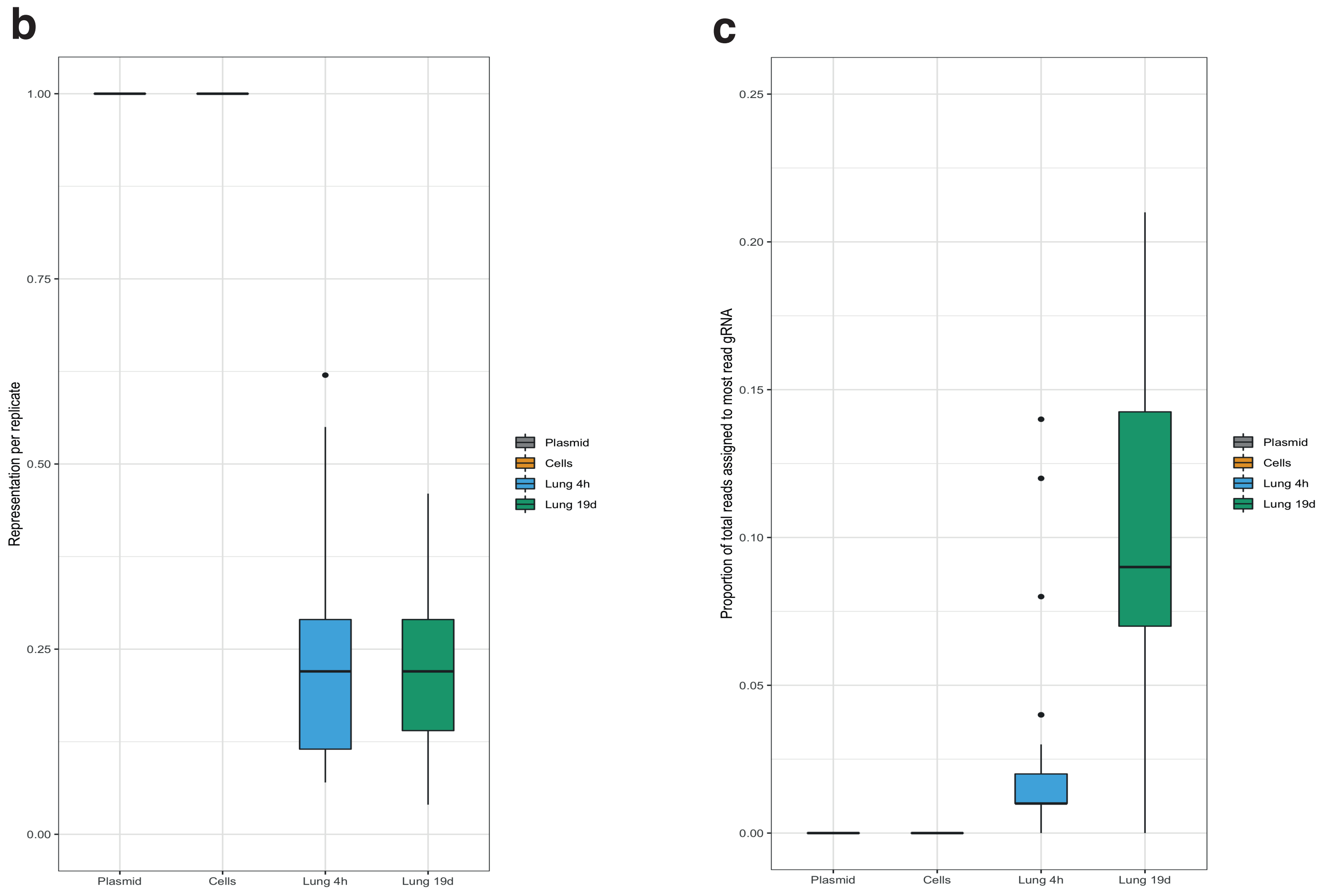

Supplementary Figure 1. Analysis of the gRNAs present in the plasmid library, cells and lungs of mice. **(a)** A breakdown of the read statistics of the screen for the 4 different categories of library replicates: plasmid, cells (500x coverage), lungs (4 hours) and lungs (19 days). **(b)** Representation of the libraries (per replicate) for each sample type, showing the lung samples with only ~20% of the total number of gRNAs present. **(c)** Proportion of total reads assigned to the gRNA with the most reads present in each sample type, showing the 19 day lung samples have enrichment of a small number of gRNAs. The lower and upper hinges correspond to the first and third quartiles (the 25th and 75th percentiles). The lower/upper whisker extend from the hinge to the smallest/largest value within 1.5-fold of the IQR. Data points outside this range are plotted individually.

Supplementary Figure 2

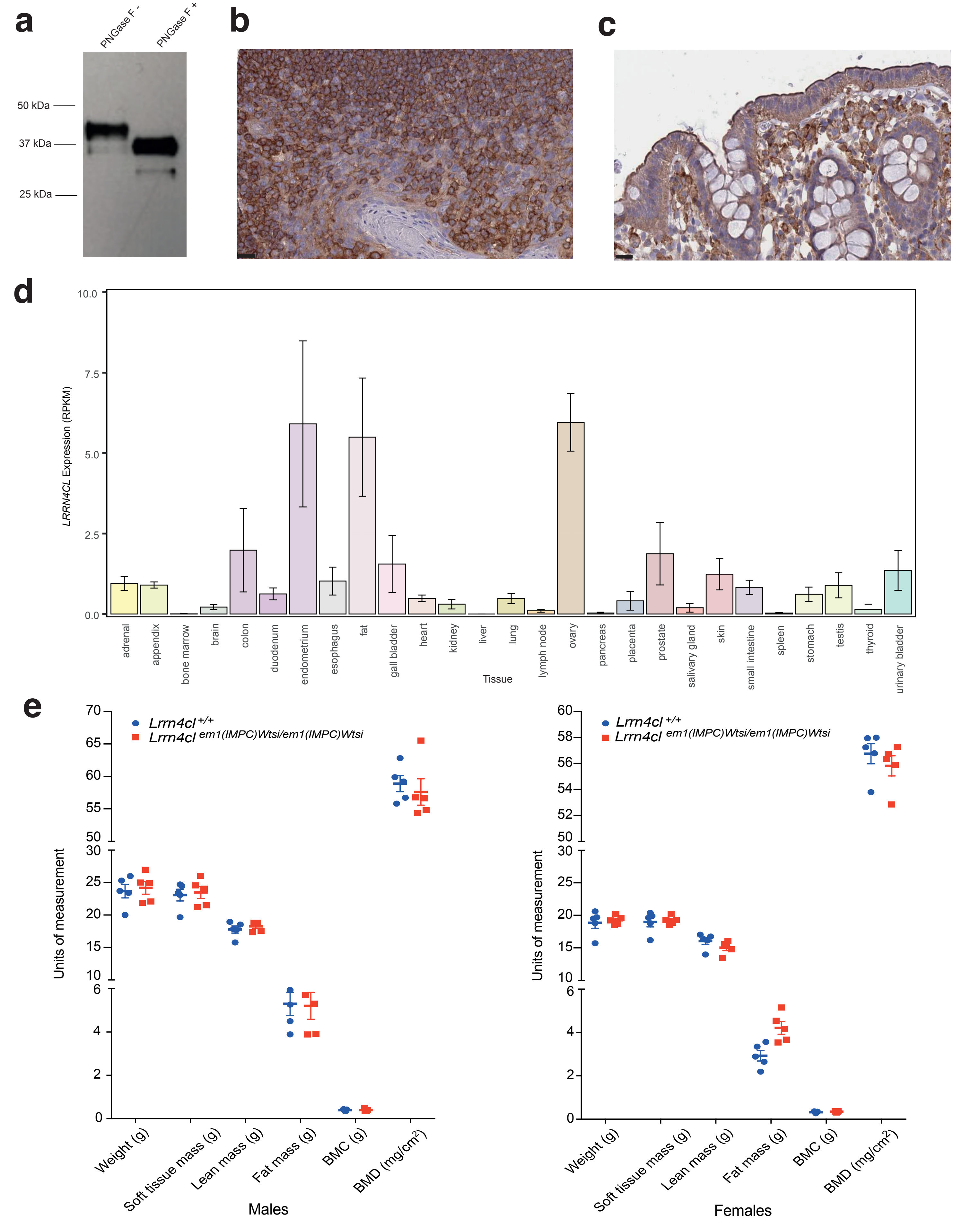

Supplementary Figure 2. Glycosylation, subcellular location and tissue distribution of LRRN4CL. **(a)** An anti-FLAG western blot using PNGase F-digested (+) B16-F0 cell lysates expressing a FLAG/streptavidin-tagged *Lrrn4cl* cDNA (LRRN4CL-FSA) showing a gel-shift compared to mock digested (-) LRRN4CL-FSA cell lysates. **(b-c)** Images of anti-LRRN4CL immunohistochemical staining of normal human tissues from The Human Protein Atlas (https://www.proteinatlas.org). Tonsil from a 27-year old male (b) and colon from a 75-year old male (c). Scale bar is 20 um. **(d)** Tissue RPKM expression of *LRRN4CL* from the NCBI BioProject database (human data from PRJEB4337[12]). Error bars show RPKM ± 1 SD. **(e)** Phenotyping results for *Lrrn4cl*-deficient mice (*Lrrn4cl*$^{em1(IMPC)Wtsi/em1(IMPC)Wtsi}$) and sex- and age-matched wildtype control (*Lrrn4cl*$^{+/+}$) mice. Each symbol represents a mouse and the bars represent mean ± SEM. BMC: bone mineral content, BMD: bone mineral density.

Supplementary Figure 3

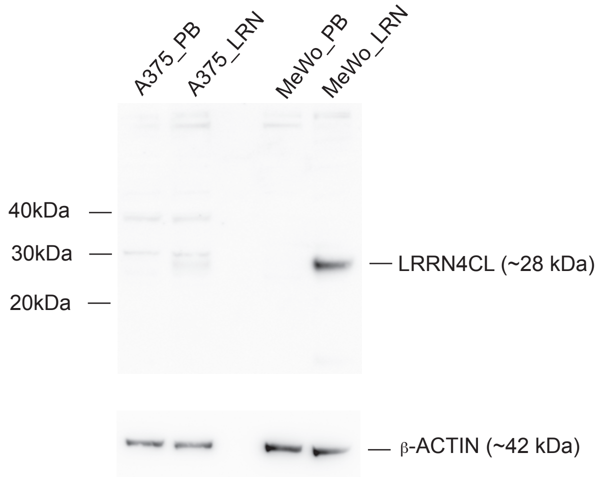

Supplementary Figure 3. Western blot of LRRN4CL expression in human melanoma cell lines. Western blot of whole cell lysates from human melanoma cell lines, A375 and MeWo, that had been stably transfected with LRRN4CL cDNA (LRN) or empty vector (PB) using an anti-LRRN4CL antibody. Anti-β-actin antibody was used as a loading control.

Supplementary Figure 4

a

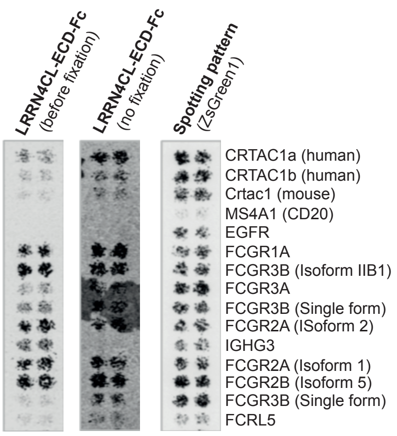

b

	Median AF647 Fluorescence (Fold change from ZsGreen-only transfected HEK293s)															
	Human CRTAC1α NM_018058				Human CRTAC1ß NM_001206528				Mouse Crtac1 NM_145123				CD20		HEKs (ZsGreen-only transfected)	
	n=1		n=2		n=1		n=2		n=1		n=2					
	Median	Fold	Median	Fold	Median	Fold	Median	Fold	Median	Fold	Median	Fold	Median	Fold	Median	
PBS + AF647 anti-hIgG Fc	986	1.2	1070	1.3	912	1.1	887	1.1	791	0.9	812	1.0	885	1.1	888	785
LRRN4CL-ECD-Fc (10µg/ml) + AF647 anti hIgG Fc	11206	8.5	10661	8.1	6556	5.0	6812	5.2	3437	2.6	3806	2.9	1157	0.9	1305	1331
Rituximab (1µg/ml) + AF647 anti-hIgG Fc	978	1.2	1019	1.2	971	1.2	912	1.1	819	1.0	786	0.9	119022	142	846	834

c

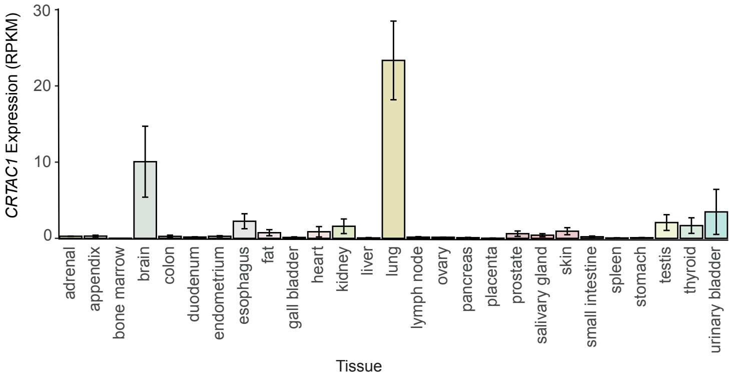

d

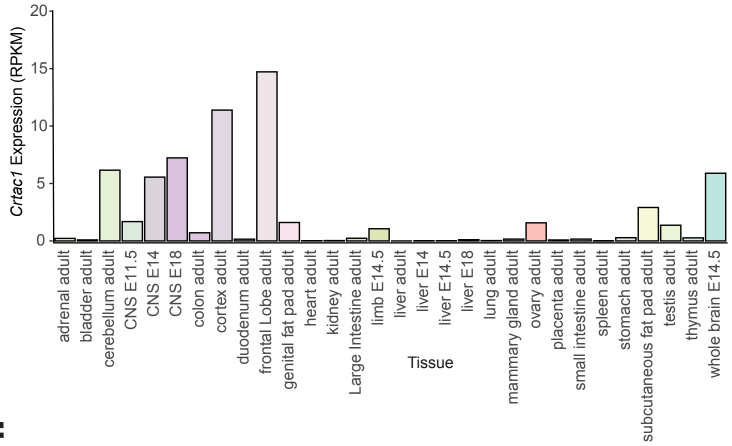

e

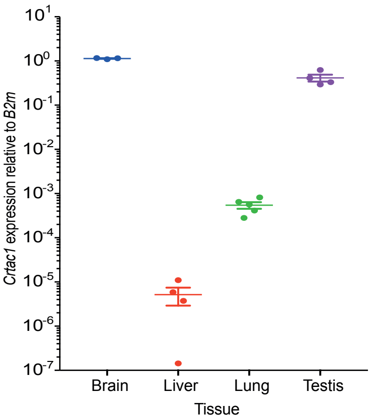

f

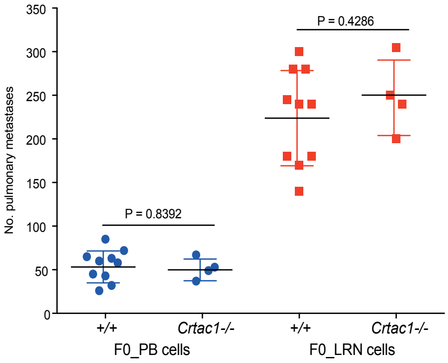

Supplementary Figure 4. Identification of CRTAC1 as a protein binding partner of LRRN4CL. **(a)** Results of the cell microarray assay showing that the LRRN4CL extracellular domain-Fc-tagged fusion protein (LRRN4CL-ECD-Fc) binds to the HEK293 cells expressing *CRTAC1* cDNA (human *CRTAC1a*, human *CRTACb* or mouse *Crtac1*) when added before fixation of the cells or when no fixation was used. Fc domain-mediated interactions are also observed with various members of the FCGR1-3 family. Key to spotting pattern showing ZsGreen1 expression which is co-expressed in the vector. MS4A.1/CD20 was the experimental control and EGFR was the transfection control. **(b)** Results of the FACS quantitation (median values) for assessing the binding between the LRRN4CL-ECD-Fc and *CRTAC* cDNA expressed in HEK293 cells (performed in duplicate). **(c)** Expression levels (RPKM) of *CRTAC1* in human tissues (data from NCBI BioProject Accession: PRJEB4337[12]). **(d)** Expression levels of *Crtac1* in mouse tissues (RPKM ± SD; data from NCBI BioProject Accession: PRJNA66167[47]). **(e)** qPCR on mouse RNA showing *Crtac1* expression levels (normalised to B2M) in the liver, lung and testis of wildtype mice (each symbol represents a mouse). **(f)** Pulmonary metastasis counts for *Lrrn4cl*-over-expressing B16-F0 melanoma cells (F0_LRN) and control cells (F0_PB) tail vein administered to wildtype (+/+) and *Crtac1* null (*Crtac1*$^{-/-}$) mice after 10 days. Each symbol represents a mouse, the bars represent mean ± SD, and statistics were performed using a Mann-Whitney t-test.

Supplementary Figure 5

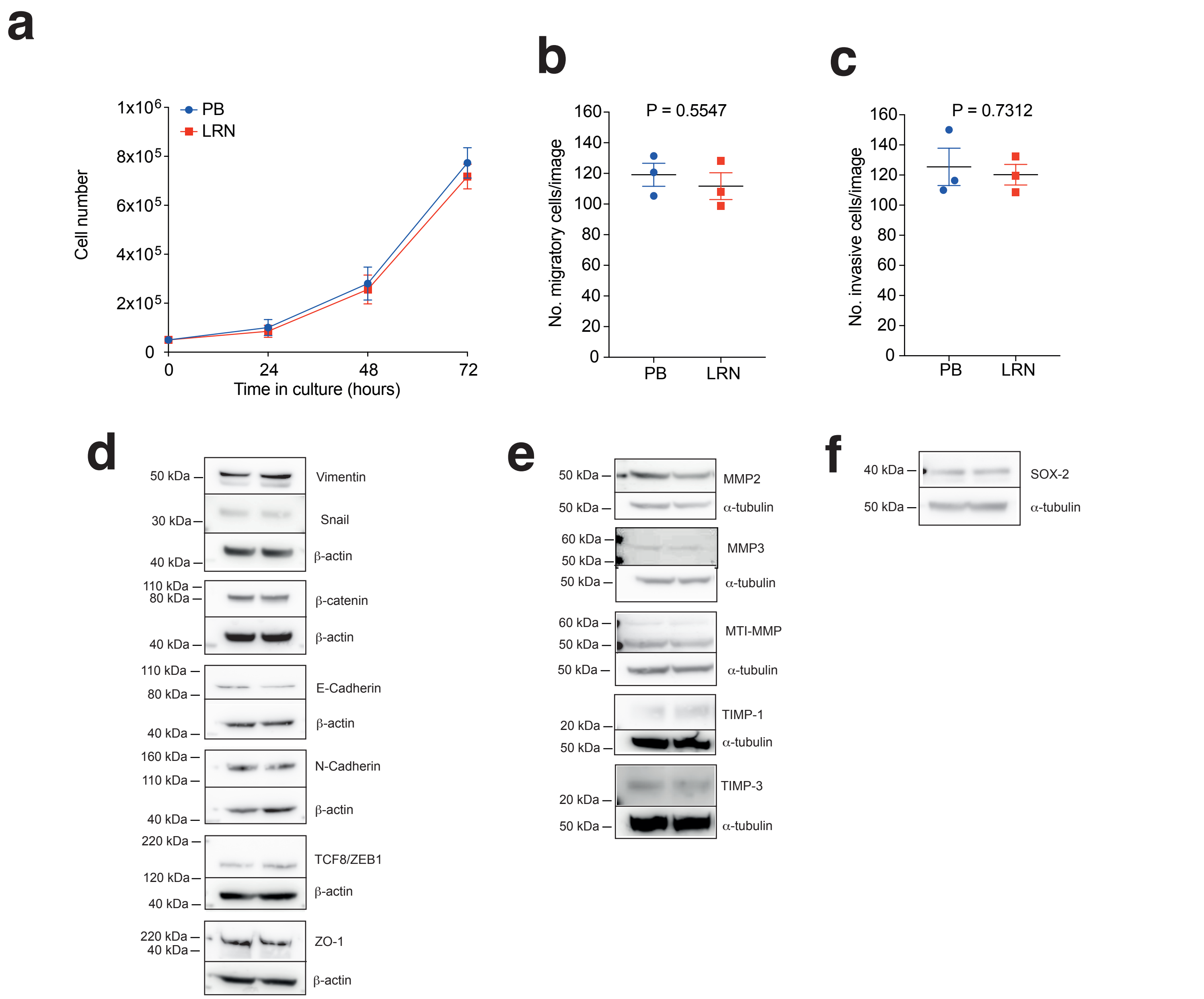

Supplementary Figure 5. *In vitro* assays to assess the metastatic capabilities of LRRN4CL-overexpressing melanoma cells. **(a)** Cell counts determined by trypan blue exclusion over a period of 72 hours for A375 cells stably transfected with a vector carrying *LRRN4CL* cDNA (LRN) or empty vector control (PB). **(b)** Cell migration of A375 PB or LRN cells in transwell chambers for 18 hours with the use of 10% FCS as a chemoattractant. Cells within three independent fields of vision were counted per transwell membrane. **(c)** Cell invasion of A375 PB or LRN cells. Conducted as for migration but with the use of Matrigel coated transwell chambers for 24 hours. For (a-c) data shown are from a single experiment (performed in technical triplicate) that is representative of 4 independent experiments (for cell growth assay and migration assay) or 3 independent experiments (for invasion assays). Data are presented as mean ± SEM, with statistical analysis performed using an unpaired t-test. **(d-f)** Western blots of key proteins involved in: (d) epithelial-mesenchymal transition, (e) matrix remodelling, and (f) pluripotency (specifically showing transcription factor SOX2, as OCT-4 and NANOG were not detected). β-actin or α-tubulin were used as loading controls. Data shown are from a single experiment that is representative of 2 independent experiments.

Supplementary Figure 6

a

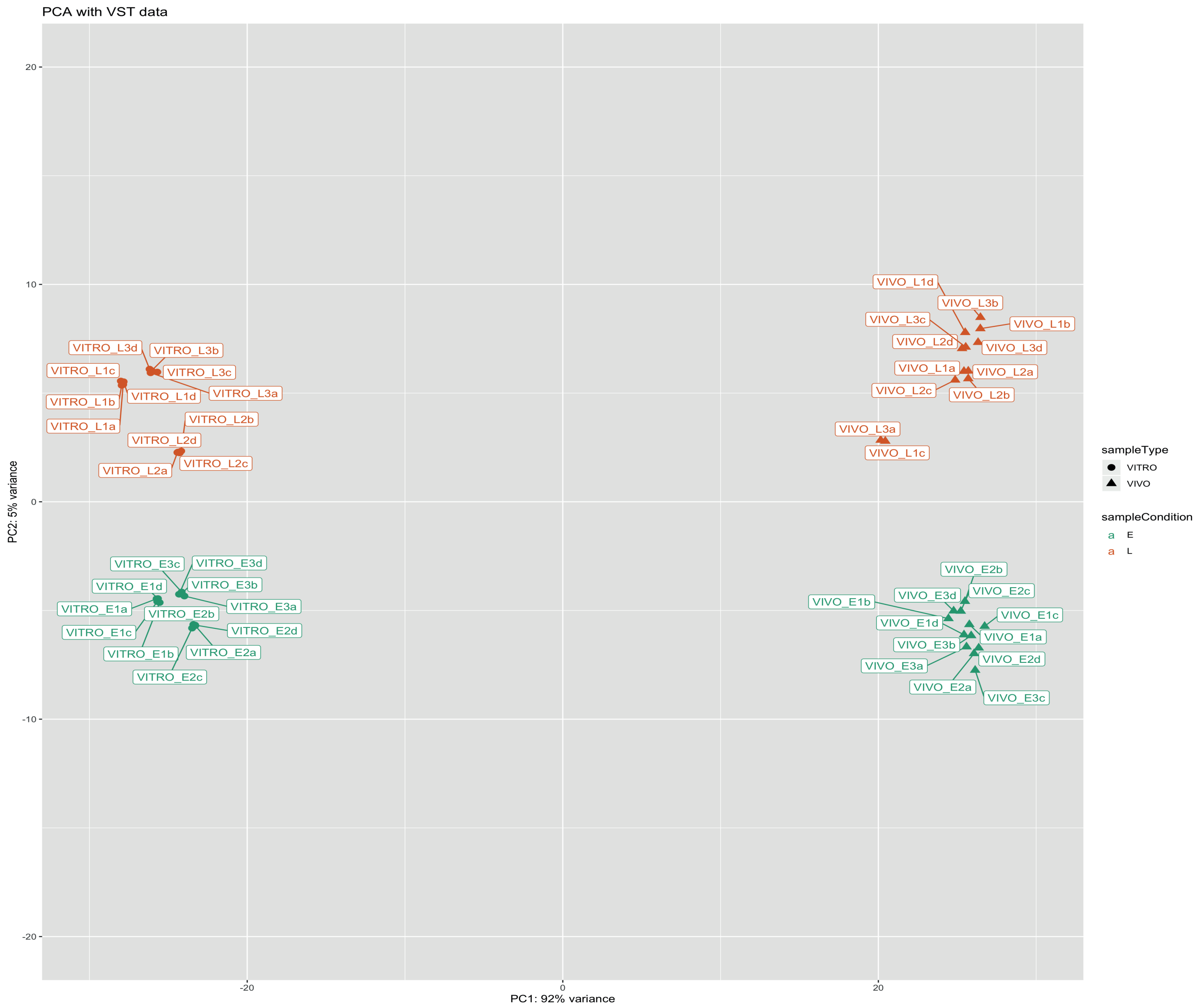

b

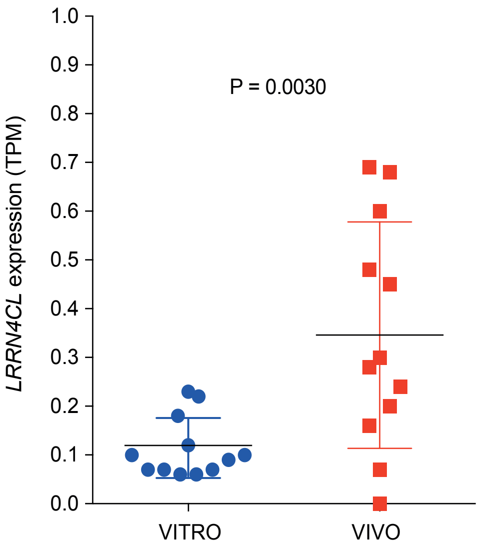

Supplementary Figure 6. Analysis of the RNAseq samples. **(a)** Principal component analysis (PCA) of the 48 samples that underwent RNAseq analysis to identify any outliers. Control cells ('E') are shown in green and LRRN4CL over-expressing cells ('L') are shown in orange. Cells that were cultured in vitro ('VITRO') are represented as circles and cells that were from the lungs of mice ('VIVO') are represented as triangles. **(b)** Expression values (TPM) for *LRRN4CL* in the control cells ('E') in the VITRO and VIVO cohorts. Each symbol represents an individual sample, the bars represent mean ± SD, and statistics were performed using an unpaired two-tailed t-test.

Supplementary Figure 7

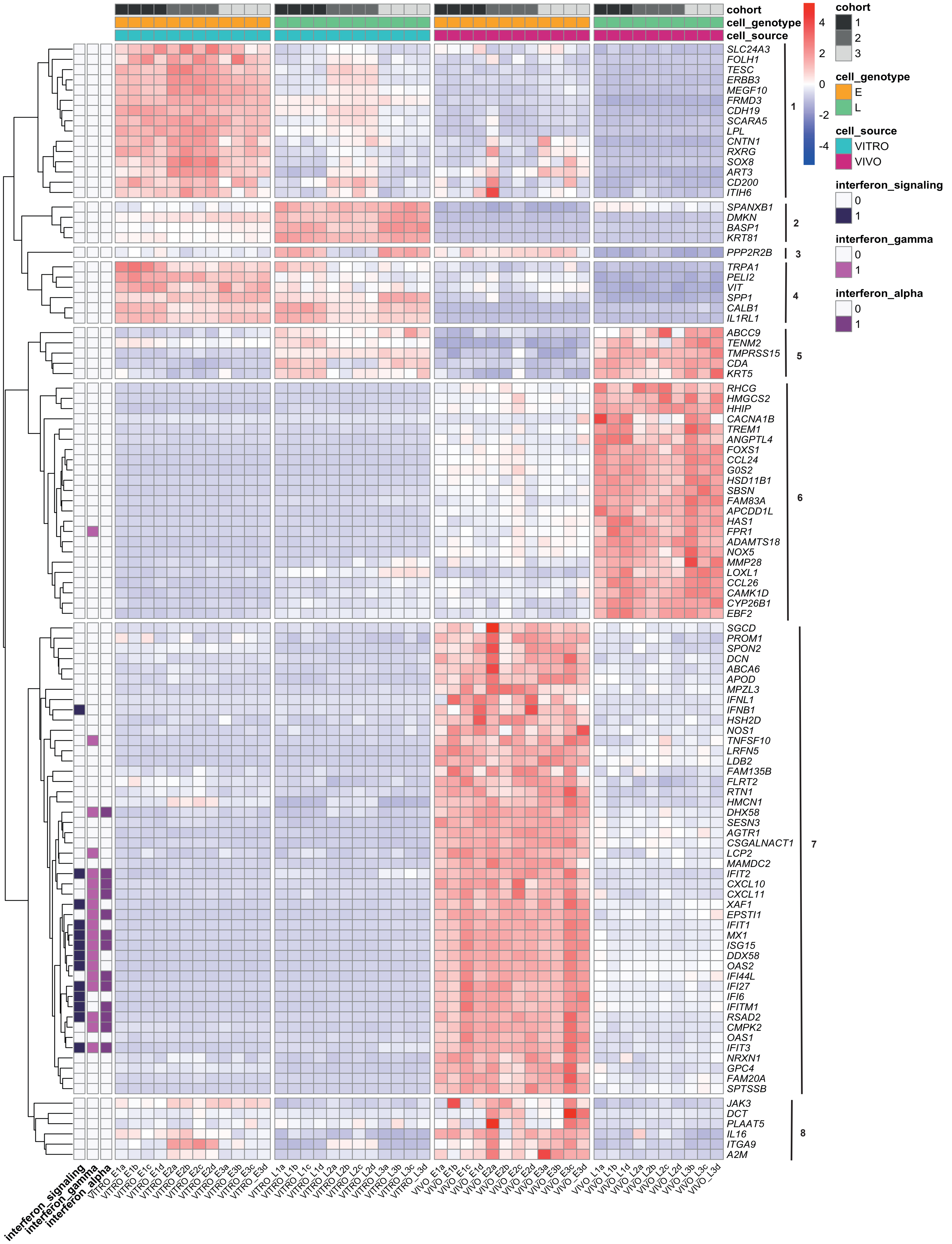

Supplementary Figure 7. *In vitro* and *in vivo* differentially expressed genes between A375 melanoma cells expressing LRRN4CL or empty vector. Heatmap of the differentially expressed genes (DEGs) between A375 melanoma cells over-expressing LRRN4CL (L) and A375 cells over-expressing the empty vector (E) that were growing *in vitro* (VITRO) or in the lungs of mice for 21 days (VIVO). Only 'protein-coding' genes are shown (9/115 DEGs were non-protein coding, thus only 106 genes shown). Three independent experiments were performed (cohort 1-3) with 4 samples (a-d) per experiment per cell line (two samples are not shown on the heatmap as they were outliers on the PCA as shown in Supplementary Fig. 6a). The black bars and numbers on the right-hand side of the heatmap signify the 8 individual clusters. Genes were scored as differentially expressed if they had a log2 fold change ≤-1 or ≥+1 with a Padj value of <0.01. Individual genes within gene set enrichment analysis (GSEA)-identified significantly downregulated pathways are shown (interferon_signaling, interferon_gamma, interferon_alpha).

Supplementary Figure 8

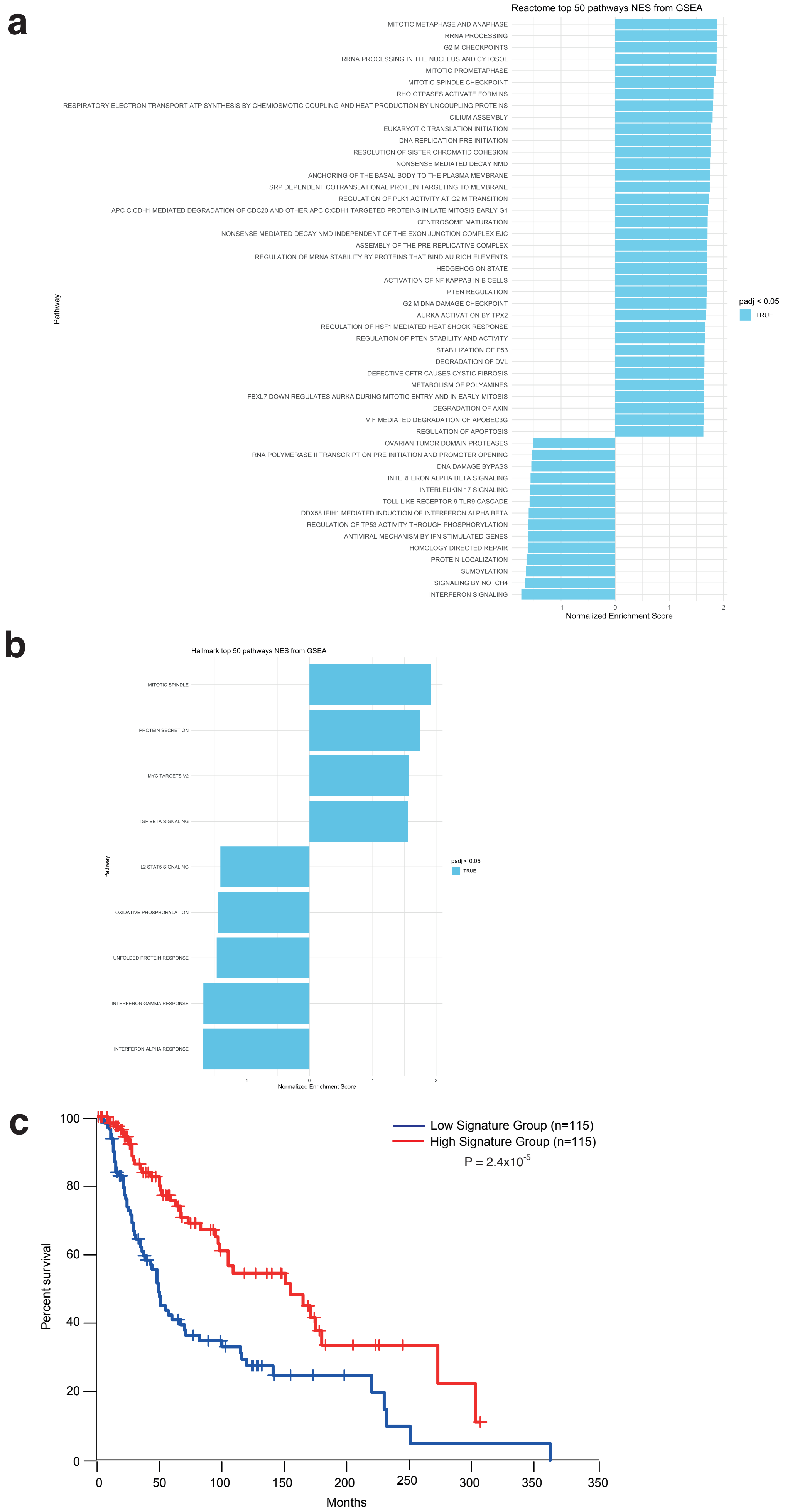

Supplementary Figure 8. Gene-set enrichment analysis on the differentially expressed genes between A375 melanoma cells expressing *LRRN4CL* or empty vector. **(a)** Reactome gene-set enrichment analysis (GSEA) on the 115 differentially expressed genes (DEGs) from the RNAseq analysis showing the top 50 pathways (P<0.05). (b) Hallmark GSEA on the 115 DEGs from the RNAseq analysis showing the top 50 pathways (P<0.05). **(b)** Hallmark GSEA on the 115 DEGs from the RNAseq analysis showing the pathways where P<0.05. **(c)** Kaplan-Meier curves for survival in melanoma patients from TCGA dataset expression levels of the 13 DEGs from the Reactome GSEA 'interferon signaling' (used as a 'signature group'; expression levels as upper or lower 25th percentile; Cox Log-rank P value).

Supplementary Figure 9

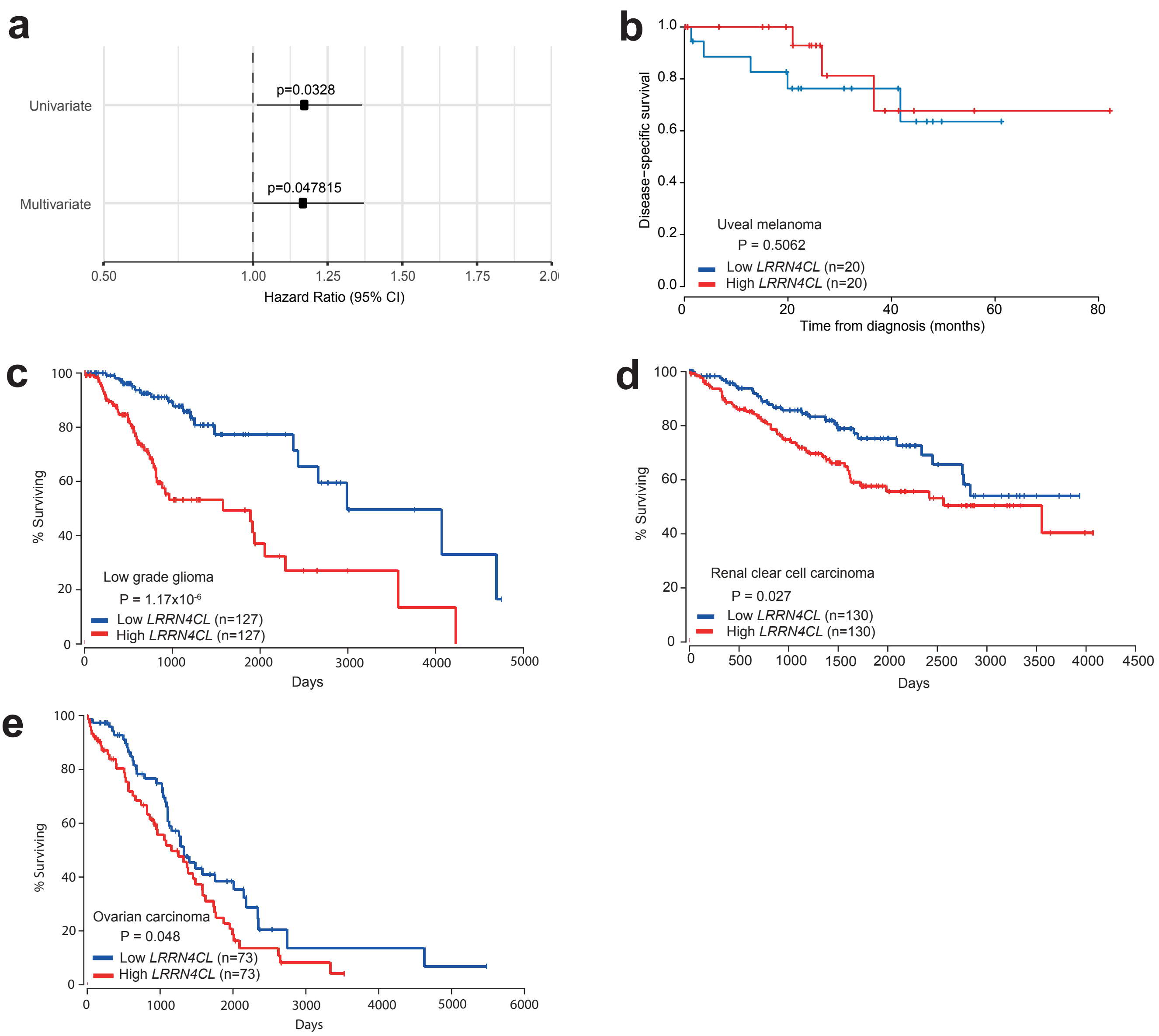

Supplementary Figure 9. Correlation of *LRRN4CL* expression levels with survival in cancer patients. **(a)** A forest plot for progression-free survival in the AVAST-M dataset[15], showing the hazard ratio (HR) (95% CI) and p-values reported in both univariate and multivariate analyses when standardised *LRRN4CL* expression scores were used as a continuous predictor in Cox regression models. Multivariate correction was undertaken for stage, sex, age, number of involved regional lymph nodes, ECOG (Eastern Cooperative Oncology Group) Performance Status and adjuvant treatment. **(b)** Kaplan-Meier curves for survival in uveal melanoma patients from the TCGA dataset. Expression levels of *LRRN4CL* as lower 25th percentile (‘low *LRRN4CL*’) or upper 25th percentile (‘high *LRRN4CL*’). Cox Log-rank P value (univariate, as insufficient data to correct for age and sex). **(c-e)** Kaplan-Meier curves for survival in cancer patients from the TCGA dataset. Expression levels of *LRRN4CL* as lower 25th percentile (‘low *LRRN4CL*’) or upper 25th percentile (‘high *LRRN4CL*’). Cox Log-rank P value, age- and sex- adjusted. The numbers in brackets on each graph indicate the number of patient samples per group.

Supplementary Figure 10

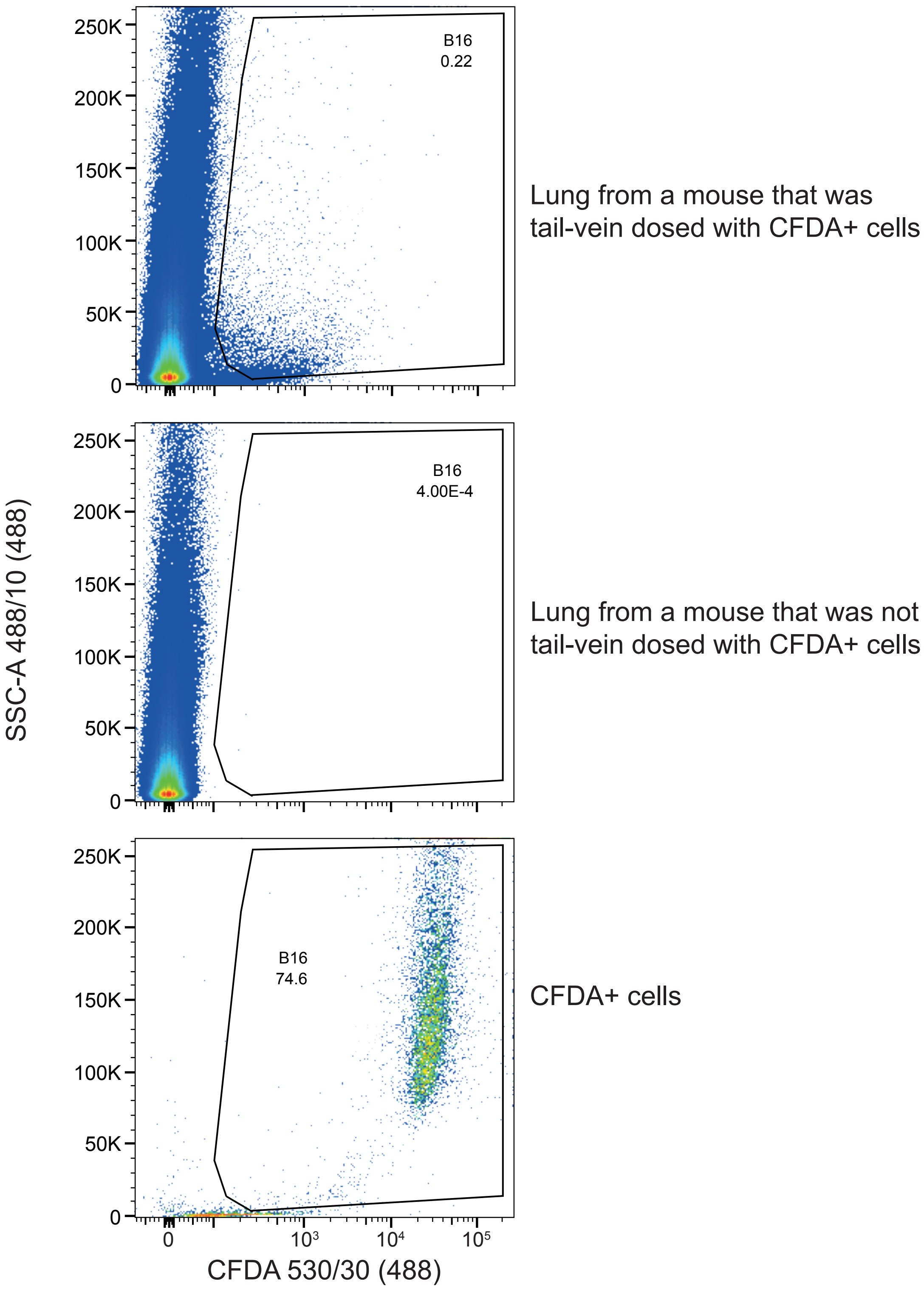

Supplementary Figure 10. Example of the gating strategy used to identify CFDA+ tumour cells in the lungs.

<u>Supplementary Table 1</u>. Top 15 results of the 'percentile ranking' method.

The gRNAs are ranked by the number of mice at day 19 in which the gRNA was found in the 98th percentile (out of a total of 35 mice) ["98%_19d" value"], followed by their Z-score ["Z_score_19d"].

gRNAs were only listed as a 'hit' if present in >10 mice at day 19

("n_19d" is the number of mice at day 19 in which the gRNA was found to be present).

**sgRNA name (from the library)**	**98%_19d**	**Z_score_19d**	**n_19d**
Lrrn4cl_+_8850757.23-P1P2	4	3.19	17
Slc4a3_+_75546398.23-P1P2	3	3.92	20
Tmem194b_+_52630698.23-P1P2	2	2.97	23
Zmynd12_+_119422735.23-P1P2	2	2.09	19
Fut2_-_45666416.23-P1P2	2	2.09	14
Smco3_+_136835494.23-P1P2	2	2.05	22
Tango6_+_106683055.23-P1P2	2	2.01	14
Cd226_+_89196992.23-P1	2	1.81	17
Gpr27_+_99692177.23-P1P2	2	1.78	26
Olfr1360_+_21675377.23-ENSMUST00000077843.3	2	1.67	18
Gpr75_-_30885367.23-P1P2	2	1.67	21
Olfr558_-_102702083.23-P1P2	2	1.59	24
Olfr878_-_37918336.23-ENSMUST00000086061.3	2	1.52	19
Olfr924_-_38848019.23-ENSMUST00000072977.4	2	1.50	22
Mla0_-_29697693.23-P1P2	2	1.48	18

<u>Supplementary Table 2</u>. Top 15 results of the JACKS analysis.
The genes are ranked by their 'effect' size' at 19 days minus that at 4 hours ("m19d_minus_m4h" value).
*Slc22a30 was discounted as there is no human ortholog [1].
Abbreviations: m, effect size of the gene; s, standard deviation; 4h, the lungs collected 4 hours after dosing; 19d, the lungs collected 19 days after dosing.

**Gene**	**m_4h**	**s_4h**	**m_19d**	**s_19d**	**m19d_minus_m4h**
Lrrn4cl	0.692	0.827	1.237	0.718	0.545
*Slc22a30**	0.828	2.894	1.316	2.325	0.488
Tm4sf19	0.240	0.767	0.625	0.637	0.385
Kcnd1	0.200	0.610	0.583	0.496	0.382
Olfr323	-0.116	0.549	0.224	0.446	0.340
Tmem54	0.045	0.846	0.369	0.675	0.324
Kcna5	0.079	0.824	0.385	0.653	0.306
Rhbdd2	0.070	0.942	0.368	0.761	0.298
Rxfp2	0.109	0.733	0.401	0.603	0.292
Scn8a	0.352	0.832	0.642	0.684	0.291
Pcdhb2	0.288	0.829	0.577	0.665	0.289
Camsap2	0.563	0.809	0.851	0.671	0.287
Olfr1396	0.316	0.830	0.585	0.680	0.269
St6galnac4	0.143	0.784	0.410	0.622	0.267
Olfr220	0.001	0.861	0.263	0.688	0.262

<u>Supplementary References</u>

1. Wu, W. et al. Analysis of a large cluster of SLC22 transporter genes, including novel USTs, reveals species-specific amplification of subsets of family members. *Physiol. Genomics* **38**, 116–124 (2009).
