# Supplementary material for: CRISPR activation screen in mice identifies novel membrane proteins enhancing pulmonary metastatic colonisation
Source: Commun Biol. 2021 Mar 23;4:395. doi: 10.1038/s42003-021-01912-w (PMC7987976; doi:10.1038/s42003-021-01912-w)
Supplement: Supplementary file 3 — Description of Additional Supplementary Files [file 42003_2021_1912_MOESM3_ESM.pdf]

# Description of Additional Supplementary Files

**File name:** Supplementary Data 1

**Description:** Results of the RNAseq experiment showing differentially expressed genes in A375_LRN cells in the in vivo context. Genes on this list were differentially expressed in VIVO_L samples versus VIVO_E samples, and not found as differentially expressed in VITRO_L samples versus VITRO_E samples (with a Padj of <0.01 and a $Log_2FC$ of ≤-1 or ≥1).

Abbreviations: $Log_2FC$, $Log_2$ fold change; Padj, adjusted P value.

**File name:** Supplementary Data 2

**Description:** Source data for the main figures.
