# Supplementary material for: CRISPR activation screen in mice identifies novel membrane proteins enhancing pulmonary metastatic colonisation
Source: Commun Biol. 2021 Mar 23;4:395. doi: 10.1038/s42003-021-01912-w (PMC7987976; doi:10.1038/s42003-021-01912-w)
Supplement: Supplementary file 6 — Reporting Summary [file 42003_2021_1912_MOESM6_ESM.pdf]

nature research

Corresponding author(s): David J. Adams

Last updated by author(s): 23.12.2020

Reporting Summary

Nature Research wishes to improve the reproducibility of the work that we publish. This form provides structure for consistency and transparency in reporting. For further information on Nature Research policies, see our Editorial Policies and the Editorial Policy Checklist.

Statistics

For all statistical analyses, confirm that the following items are present in the figure legend, table legend, main text, or Methods section.

n/a	Confirmed	
☐	☒	The exact sample size (*n*) for each experimental group/condition, given as a discrete number and unit of measurement
☐	☒	A statement on whether measurements were taken from distinct samples or whether the same sample was measured repeatedly
☐	☒	The statistical test(s) used AND whether they are one- or two-sided<br>*Only common tests should be described solely by name; describe more complex techniques in the Methods section.*
☐	☒	A description of all covariates tested
☐	☒	A description of any assumptions or corrections, such as tests of normality and adjustment for multiple comparisons
☐	☒	A full description of the statistical parameters including central tendency (e.g. means) or other basic estimates (e.g. regression coefficient) AND variation (e.g. standard deviation) or associated estimates of uncertainty (e.g. confidence intervals)
☐	☒	For null hypothesis testing, the test statistic (e.g. *F*, *t*, *r*) with confidence intervals, effect sizes, degrees of freedom and *P* value noted<br>*Give P values as exact values whenever suitable.*
☒	☐	For Bayesian analysis, information on the choice of priors and Markov chain Monte Carlo settings
☐	☒	For hierarchical and complex designs, identification of the appropriate level for tests and full reporting of outcomes
☒	☐	Estimates of effect sizes (e.g. Cohen's *d*, Pearson's *r*), indicating how they were calculated

Our web collection on statistics for biologists contains articles on many of the points above.

Software and code

Policy information about availability of computer code

Data collection	Sequence data was generated on the Illumina platform. Raw sequence reads were released into the European Nucleotide Archive under the accession number: ERP123242 and the European Genome-phenome Archive (EGA) accession number EGAD00001006249.
Data analysis	RNA sequencing data: DESeq2 and the R package fgsea (version 1.12.0) with the MSigDB (version 7.0); CRISPR analysis: MaGeCK (version 0.5.8) and JACKS (March 2018); Flow cytometry: FlowJo 10.7; survival analysis: in R using the survival package (version 3.1-12) and coxph function of the survival package (v2.2-3).

For manuscripts utilizing custom algorithms or software that are central to the research but not yet described in published literature, software must be made available to editors and reviewers. We strongly encourage code deposition in a community repository (e.g. GitHub). See the Nature Research guidelines for submitting code & software for further information.

Data

Policy information about availability of data

All manuscripts must include a data availability statement. This statement should provide the following information, where applicable:

- Accession codes, unique identifiers, or web links for publicly available datasets
- A list of figures that have associated raw data
- A description of any restrictions on data availability

* Files for the CRISPR sequencing data are available under the European Nucleotide Archive (ENA) accession number: ERP123242, and files for the RNAseq data are available under the European Genome-phenome Archive (EGA) accession number EGAD00001006249.
* GEPIA 2 [http://gepia2.cancer-pku.cn/#survival].

Field-specific reporting

Please select the one below that is the best fit for your research. If you are not sure, read the appropriate sections before making your selection.

☒ Life sciences ☐ Behavioural & social sciences ☐ Ecological, evolutionary & environmental sciences

For a reference copy of the document with all sections, see nature.com/documents/nr-reporting-summary-flat.pdf

Life sciences study design

All studies must disclose on these points even when the disclosure is negative.

Sample size	Sample sizes for each experiment were based on previous experiences (van der Weyden et al., 2017; Nature; PMID: PMID: 28052056)
Data exclusions	No data were excluded.
Replication	All experiments were replicated at least 2-3 times and on all occasions the results were concordant.
Randomization	For most experiments, random allocation to dosing group was achieved through the process of Mendelian inheritance, with age- and sex-matched mice being selected across different litters and matings (to minimize potential litter and/or cage effects).
Blinding	Counting of the number of metastases were counted and the growth of tumours was measured with the researcher blind to treatment (i.e., cell line used). All analysis of H&E-stained slides by the pathologist was done in a blinded manner. All samples analysed using FlowJo 10.7 were done in a treatment blind manner.

Reporting for specific materials, systems and methods

We require information from authors about some types of materials, experimental systems and methods used in many studies. Here, indicate whether each material, system or method listed is relevant to your study. If you are not sure if a list item applies to your research, read the appropriate section before selecting a response.

Materials & experimental systems

n/a	Involved in the study	
☐	☒	Antibodies
☐	☒	Eukaryotic cell lines
☒	☐	Palaeontology and archaeology
☐	☒	Animals and other organisms
☒	☐	Human research participants
☒	☐	Clinical data
☒	☐	Dual use research of concern

Methods

n/a	Involved in the study	
☒	☐	ChIP-seq
☐	☒	Flow cytometry
☒	☐	MRI-based neuroimaging

Antibodies

Antibodies used	Epithelial-Mesenchymal Transition (EMT) Antibody Sampler Kit (#9782, Cell Signaling). Matrix Remodeling Antibody Sampler Kit (#73959, Cell Signaling). StemLight™ Pluripotency Transcription Factor Antibody Kit (#9093, Cell Signaling). LRRN4CL antibody – middle region (#OAAB08983, Aviva Systems Biology), β-Actin (8H10D10) mouse monoclonal Antibody (#3700, Cell Signaling), α-Tubulin Antibody (#2144, Cell Signaling), and mouse monoclonal anti-FLAG® M2-Peroxidase (#A8592, Sigma). AlexaFluor647-conjugated goat anti-human IgG Fcγ pAbs (#109-605-098, Jackson ImmunoResearch).
Validation	Cell Signaling (CST) antibodies: "To ensure our antibodies will work in your experiment, we adhere to the Hallmarks of Antibody Validation™, six complementary strategies that can be used to determine the functionality, specificity, and sensitivity of an antibody in any given assay. CST adapted the work by Uhlen, et. al., ("A Proposal for Validation of Antibodies." Nature Methods (2016)) to build the Hallmarks of Antibody Validation, based on our decades of experience as an antibody manufacturer and our dedication to reproducible science" (https://www.cellsignal.co.uk/contents/our-approach/cst-antibody-validation-principles/ourapproach-validation-principles).<br>AlexaFluor647-conjugated goat anti-human IgG Fcγ pAbs (#109-605-098, Jackson ImmunoResearch): "Based on immunoelectrophoresis and/or ELISA, the antibody reacts with the Fc portion of human IgG heavy chain but not with the Fab portion of human IgG. No antibody was detected against human IgM or IgA, or against non-immunoglobulin serum proteins. The antibody has been tested by ELISA and/or solid-phase adsorbed to ensure minimal cross-reaction with bovine, horse and mouse serum proteins, but it may cross-react with immunoglobulins from other species" (https://www.jacksonimmuno.com/catalog/products/109-605-098/Goat-Human-IgG-Fc-Alexa-Fluor-647).<br>Mouse monoclonal anti-FLAG® M2-Peroxidase (#A8592, Sigma): this product has been cited in 936 papers (https://www.sigmaaldrich.com/catalog/product/sigma/A8592?lang=en&region=GB).<br>LRRN4CL antibody – middle region (#OAAB08983, Aviva Systems Biology): this antibody was purified through a protein A column,

followed by peptide affinity purification. It is designed for Western blotting and immunohistochemistry (https://www.avivasysbio.com/lrrn4cl-antibody-center-region-oaab08983.html).

Eukaryotic cell lines

Policy information about cell lines

Cell line source(s)	The mouse melanoma B16-F0 cell line was from ATCC and the mouse melanoma B16-BL6 cell line was from the University of Texas, MD Anderson Cancer Center. The human melanoma A375 and MeWo cell lines were purchased from ATCC. The mouse cell lines that were gifts from the laboratories that generated them: the mouse melanoma YUMM1.7 cell line was from M. Bosenberg (Yale University School of Medicine, USA) [Meeth et al., 2016], the mouse bladder cancer MB-49 cell line was from A. Hegele (Philipps University of Marburg, Germany) [Summerhaynes and Franks, 1979], the metastatic mouse colorectal MC-38 cell line was a gift from L. Borsig (University of Zurich, Switzerland) [Borsig et al., 2002], the metastatic mouse mammary cancer EO771.LMB cell line was a gift from R. L. Anderson (Peter MacCallum Cancer Centre, Australia) [Johnstone et al., 2015], and the metastatic HCmel12–mCherry melanoma cell line was a gift from T. Tuting (University Hospital Magdeburg, Germany) [Bald et al., 2014].
Authentication	The mouse melanoma B16-F0 cell line and the mouse melanoma B16-BL6 cell line were authenticated by whole genome and transcriptome sequencing. The human melanoma A375 and MeWo cell lines were verified by STR profiling. The mouse cell lines that were gifts from the laboratories that generated them (melanoma HCmel12, colorectal cancer MC-38, breast cancer EO771, melanoma YUMM1.7 and bladder cancer MB-49 cell lines) were not validated.
Mycoplasma contamination	The cell lines were screened for the presence of mycoplasma and mouse pathogens (at Charles River Laboratories, USA).
Commonly misidentified lines (See ICLAC register)	None were used.

Animals and other organisms

Policy information about studies involving animals; ARRIVE guidelines recommended for reporting animal research

Laboratory animals	Wildtype mice (C57BL/6NTac), immunodeficient mice (NOD.CgPrkdcscid, Il2rgtm1Wjl/SzJ), Lrrn4cl'knockout' mice (Lrrn4clem1(IMPC)Wtsi) and Crtac1 'knockout' mice (C57BL/6NTac-Crtac1em1(IMPC)H/H). Mice were 6- to 12-weeks old and both sexes were used.
Wild animals	N/A
Field-collected samples	N/A
Ethics oversight	The care and use of all mice in this study were in accordance with the Home Office guidelines of the UK and procedures were performed under a UK Home Office Project Licence (P6B8058B0), which was reviewed and approved by the Sanger Institute's Animal Welfare and Ethical Review Body.

Note that full information on the approval of the study protocol must also be provided in the manuscript.

Flow Cytometry

Plots

Confirm that:

- ☐ The axis labels state the marker and fluorochrome used (e.g. CD4-FITC).
- ☐ The axis scales are clearly visible. Include numbers along axes only for bottom left plot of group (a 'group' is an analysis of identical markers).
- ☐ All plots are contour plots with outliers or pseudocolor plots.
- ☐ A numerical value for number of cells or percentage (with statistics) is provided.

Methodology

Sample preparation	* To quantify the number of tumour cells entering the lungs, tumour cells (either control or Lrrn4cl upregulated) were fluorescently labelled with CFDA and intravenously dosed into mice. At the specified timepoints the mice were sacrificed and saline perfused to remove circulating cells. The lungs were processed in GentleMACS C tubes on a GentleMACS dissociator with program 'lung_01' in HBSS with calcium and magnesium and Liberase DL added (0.1U/ml). Samples were incubated at 37oC for 30 min then run on program 'lung_02' and DNase I added (0.1 mg/ml) before being incubated for a further 30 min at 37oC. Debris was removed by passing samples through a 30 micron strainer and analysed.<br>* For sorting of mCherry-A375 human cancer cells (control or LRRN4CL upregulated) from mouse lungs for transcriptomic analysis, lungs were isolated from saline perfused mice and processed with the human tumour dissociation kit and mouse cell depletion kit (both Miltenyi Biotec) according to the manufacturers instructions. Mouse cell depleted samples were resuspended in D-PBS with 0.5% FBS for sorting.<br>* Cell lines were analysed for BFP or mCherry expression after trypsination, quenching with complete media and resuspended in FACS buffer (D-PBS with 0.5% FBS, 2mM EDTA and 0.09% sodium azide) for analysis.<br>CRTAC-LRRN4CL binding experiments were performed by Retrogenix Ltd (https://www.retrogenix.com/).

Instrument	Samples were analysed on BD Fortessa SORP or BD LSR II instruments or sorted on BD FACS Aria IIIu. Retrogenix experiments were done on a BD Accuri (all machines from Becton Dickinson).
Software	All data was collected using BD FACSDiva and analysed with FlowJo.
Cell population abundance	For sorting to generate the mCherry expressing lines the same sort gate was used for both control and LRRN4CL overexpressing to ensure the same fluorescence intensity. Post-sort purity was checked by running the samples on the same machine and was >95%. After expansion of the cell lines (approx 10 days) the mCherry signal was confirmed on a flow analyser.<br>For transcriptomic samples due to the limited number of cells in the samples obtained from the lungs a pool of leftover samples was used to sort and this material was used for the post sort purity check on the same instrument and was >95%.
Gating strategy	* For gating mCherry+ or CFDA+ cells from the lungs, an undosed mouse lung and the parental cell lines were used as negative and positive gating controls respectively.<br>* For sorting mCherry cells (from the lungs or in vitro culture) the following gating strategy was used: singlets were identified first on an FSC-A vs FSC-H and then SSC-H vs SSC-W plot. Next a stringent cell gate was used to exclude debris or dying cells on an FSC-A vs SSC-A plot. Then for the sorting of mCherry cell lines, a histogram plot was used to select the mCherry positive population with the same gate used for both cell lines (A375_PB and A375_LRN). For the transcriptomic samples the mCherry cells were identified using a mCherry-A vs SSC-A plot.<br>* CRTAC-LRRN4CL binding experiments were performed by Retrogenix Ltd (https://www.retrogenix.com/).

☒ Tick this box to confirm that a figure exemplifying the gating strategy is provided in the Supplementary Information.
